# Early results from an Egyptian transcatheter aortic valve registry (Egy-TVR)

**DOI:** 10.1186/s43044-021-00189-y

**Published:** 2021-07-20

**Authors:** Hesham Bahaa, Yasser Sadek, Ahmad E. Mostafa, Diaa Kamal, Mahmoud Baraka, Mohammad Abdelghani, Ahmed Hassan, Ahmed Shehata, Magdy Abdelhamid, Ahmed Elguindy

**Affiliations:** 1 Department of Adult Cardiology, Aswan Heart Centre, Magdi Yacoub Foundation, Aswan, Egypt; 2 Department of Cardiology, National Heart Institute, Cairo, Egypt; 3 Department of Cardiology, Helwan University, Helwan, Egypt; 4 Department of Cardiology, Ain Shams University, Cairo, Egypt; 5 Department of Cardiology, Al-Azhar University, Cairo, Egypt; 6 Amsterdam UMC, University of Amsterdam, Amsterdam, The Netherlands; 7 Department of Cardiology, Cairo University, Cairo, Egypt; 8 National Heart and Lung Institute, Imperial College London, London, UK

**Keywords:** TAVI, Transcatheter aortic valve implantation, Egyptian, Registry, Aortic stenosis, Egy-TVR, Transcatheter valve registry

## Abstract

**Background:**

Transcatheter aortic valve implantation (TAVI) is a well-established and standard therapy for patients with symptomatic severe aortic stenosis at moderate or high risk for surgical aortic valve replacement. Recently, it has proven non-inferior in patients with low surgical risk. However, due to its high cost, the availability of TAVI is variable worldwide. Our aim was to assess the demographic and clinical characteristics and short-term and long-term outcome of those patients. A medical registry is believed to be an excellent tool to perform a field analysis of patients’ course, documenting short, intermediate, and long-term outcomes. This is the first registry for patients who underwent TAVI in Egypt.

**Results:**

Ninety-six patients were included in the study; some were retrospective, and the majority were prospective from 5 different cardiac centers from August 2012 till December 2017. The mean age of patients was 77 years SD ± 7.29; females were 52% of the patients and most of the patients were overweight (BMI 30.74, SD ± 6.83). Sixty-three percent of the patients were frail with Katz index ≤ 5. 3.5% had atrial fibrillation (AF) at presentation. General anesthesia was conducted in only 59.37% of the patients. Transfemoral access was the prevailing route of implantation (90%). The median hospital stay was 4 days. In-hospital and 30 days mortality was only 4.16%.

**Conclusion:**

TAVI outcome in Egypt appeared to be very promising with in-hospital complication, and mortality rates being comparable to international registries (4.16% vs. 4.0% in TVT registry) denoting the procedure as safe and beneficial. Establishing a national registry is critical to highlighting strength and weaknesses as well as identifying key areas for improvements.

**Supplementary Information:**

The online version contains supplementary material available at 10.1186/s43044-021-00189-y.

## Background

Aortic stenosis (AS) is known to have a long latent period that is usually followed by rapid deterioration once patients develop symptoms [1], with up to 50% mortality in the first 2 years in if left untreated [2].

For decades, surgical aortic valve replacement (SAVR) has been the gold standard therapy for symptomatic patients with severe AS. In patients with no serious comorbidities, SAVR usually carries a low operative risk of mortality [3]. However, in clinical practice, almost 30 to 50% of patients with severe AS are denied surgical replacement [4]. This is attributed to a number of reasons including advanced age, frailty, depressed left ventricular function, other organ dysfunction, or other conditions that deem those patients at high/prohibitive risk for SAVR. For decades, a less invasive treatment modality that is equally safe and effective as SAVR remained an unmet need [3].

Transcatheter aortic valve implantation (TAVI) has become a well-established modality for the treatment of severe AS. Initially introduced for patients with high or prohibitive risk for surgery, the indication for TAVI is now extending to intermediate and low surgical risk patients [5, 6].

Since its first in man in 2002, TAVI has seen exponential growth being implanted in more than 300,000 patients with an annual increase of 40% by the year 2016 [7].

Registries are believed to be an excellent tool for unselected real-world patients and provide important insight into short-, intermediate-, and long-term outcomes and can be utilized to determine cost effectiveness of various interventions [8]. In the present study, we sought to report the clinical outcomes of an all-comer Egyptian TAVI cohort operated upon by 5 Egyptian operators in 5 centers who collective created the Egy-TVR registry.

## Methods

The study included patients with symptomatic severe AS admitted for TAVI in five Egyptian centers in the period from August 2012 to December 2017. The study protocol was approved by the local ethics committee at each of the participating centers and conforms to the Declaration of Helsinki.

Each patient was provided a written informed consent for analysis of anonymized data.

Preprocedural, procedural, post-procedural, and follow-up data were collected and recorded using an electronic case report form (CRF) filled for each patient, which is then uploaded to a shared web-based database engine “REDCap”.

For all patients enrolled in the Egyptian TAVI Registry, the following data were collected and recorded: demographic data, medical history, and lab results. A copy of the electronic CRF is included in the supplementary materials. Minnesota Living with Heart Failure Questionnaire (to estimate the quality of life; QoL) were filled. Frailty Index (Katz Index of Independence in activities of daily living) was reported for all patients. Electrocardiogram ECG and echocardiography data (according to the criteria of the American Society of Echocardiography guidelines [9]) were collected and reported. Also, CT data and cardiac catheterization data were included. During the Heart Team (mainly cardiologists, cardiac surgeons, and anesthetists) decision, surgical risk was estimated using EuroScore II and the Society of Thoracic Surgeons predicted risk of mortality (STS-PROM), as well as the Katz frailty index, and procedure-specific impediments to SAVR (such as porcelain aorta, patent arterial graft adherent to the posterior chest wall). The procedural data, outcome, and complications were collected according to the Valve Academic Research Consortium-2 (VARC-2) [10]. The follow-up data was collected at 3-month and 6-month intervals, including clinical and echocardiographic outcomes. Echocardiographic data included (1) stability of the valve, (2) the presence of paravalvular leaks (PVL) and their location (in the short axis view) and degree, (3) the gradient across the prosthesis and comparing it to the baseline measurement, and (4) the degree of mitral regurgitation and comparing it to the baseline study.

All results were collected in a specially designed database, tabulated, and analyzed using SPSS (version 24). Continuous variables were summarized as mean ± standard deviation (SD), while categorical variables were summarized as frequency (proportion).

## Results

**Fig. 1:**
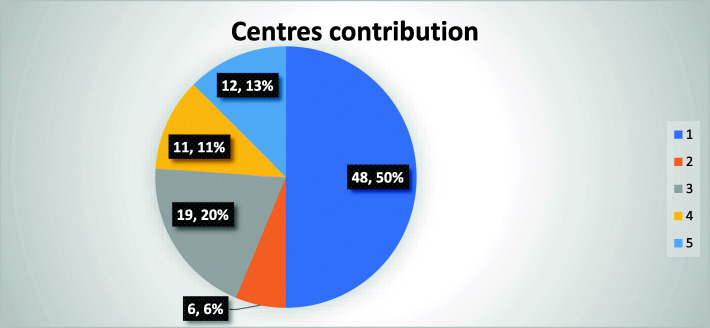
Centers contribution. (1) Aswan Heart Centre, Magdi Yacoub Foundation; (2) Galaa Military Hospital Magdi Yacoub Foundation Unit; (3) Ain Shams University Hospital; (4) Dar El fouad, October city; and (5) Andalusia Hospital

**Fig. 2:**
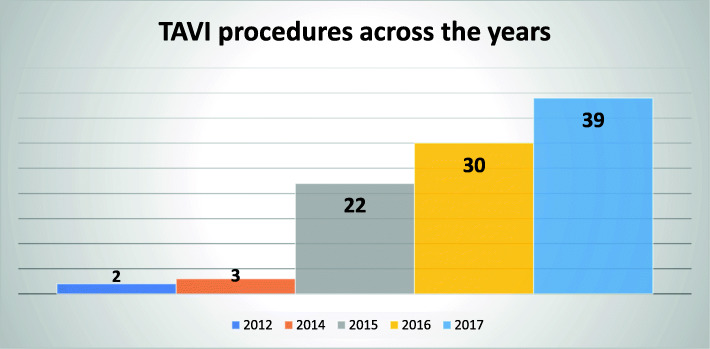
TAVI procedures across the years

The study included 96 patients from 5 centers, their contribution is shown in Fig. 1. All TAVIs were performed by interventional cardiologist, with back up surgeons on-site. Figure 2 shows the continuous increase of the performed TAVI in Egypt across the years.

Table 1 summarizes the baseline clinical characteristics of all patients. Briefly, the mean age was 77.2 ± 7.3 years, 52% of patients were female, mean STS-PROM was 5.8 ± 5.6, and 75% were severely symptomatic (NYHA III-IV). At baseline, aortic transvalvular mean gradient was 49.1 ± 14.0 mmHg, left ventricular ejection fraction was 62.8 ± 12.6%, and the prevalence of severe mitral regurgitation, tricuspid regurgitation, and pulmonary hypertension was 41%, 25%, and 21%, respectively. Other baseline echocardiographic characteristics are summarized in Table 2. Only 5 patients had bicuspid severe aortic stenosis (mean STS was 3.87% and mean age 76.4 years) and 2 patients had severe rheumatic aortic stenosis. The rest of the patients suffered severe calcific degenerative aortic stenosis.

**Table 1:** Baseline characteristics

Clinical characteristics	Mean ± (SD), n (%)
Age (years)	77.17 ±(7.29)
Female	50 (52.1%)
Weight (kg)	80.44 ±(19.2)
Height (cm)	161.56±(8.86)
BMI (kg/m^2^)	30.74 ± (6.83)
BSA (m^2^)	1.83 ± (0.23)
Diabetes	45 (46.8%)
Oral hypoglycemic	23 (24.1%)
Insulin	22 (22.9%)
Hypertension	66 (68.7%)
Ex-smoking	25 (26.0%)
Smoker	3 (3.1%)
Regular dialysis	5 (5.2%)
CrCl < 30 ml/h	17(17.7%)
NYHA III	72 (75%)
Katz index ≤ 5	61 (63.5%)
AF	13 (13.5%)
LBBB	8 (8.3%)
Paced rhythm	7 (7.3%)
Previous CABG	5 (5.20%)
Previous valve surgery	3 (3.10%)
Previous MI	10 (10.41%)
Critical pre-operative state	6 (6.2%)
STS-PROM	5.78 ± (5.6)
EuroScore II	6.4 ± (7.1)

*BMI* body mass index, *BSA* body surface area, *CrCl* creatinine clearance, *NYHA* New York Heart Association, *AF* atrial fibrillation, *LBBB* left bundle branch block, *CABG* coronary artery bypass grafting, *MI* myocardial infarction, *STS-PROM* Society of Thoracic Surgeons Predicted Risk Of Mortality

**Table 2:** Baseline echocardiographic

Echocardiography	Mean ± (SD), n (%)
LVIDd (cm)	5.04 ± (0.82)
LVIDs (cm)	3.24 ± (0.86)
EF%	62.78% ± (12.6)
Aortic annulus (mm)	22.17 ± (4.2)
Peak gradient across AV (mmHg)	80.5 ± (19.8)
Mean gradient across AV (mmHg)	49.14 ± (14.01)
Mitral regurgitation moderate or severe	39 (40.6%)
Tricuspid regurgitation moderate or severe	24 (25%)
Severe pulmonary hypertension > 60 mmHg	20 (20.83%)
Mean estimated right ventricular systolic pressure (mmHg)	44.19 ± 19.57

*LVIDd* left ventricular internal dimension in diastole, *LVIDs* left ventricular internal dimension in systole, *EF* ejection fraction, *AV* aortic valve

### CT-derived parameters

CT is fundamental for TAVI planning. In Table 3, the CT findings of enrolled patients are detailed. The left ventricular outflow tract (LVOT) region was defined as the region between the basal ring and 15 mm inferior into the left ventricle. Severe LVOT calcification was identified by calcium volume above 609 mm^3^ in the LVOT region calculated.

**Table 3:** CT aortic measurements

CT parameter	Mean (SD), n (%)
Mean annular diameter (mm)	24.23 ± (2.8)
Sinus of Valsalva (mm)	30.0 ± (5)
sinotubular junction (mm)	27.26 ± (4.5)
Left coronary height (mm)	13.5 ± (2.23)
Right coronary height (mm)	14.4 ± (2.8)
Annulus area (cm^2^)	4.39 ± (0.82)
Annulus perimeter (mm)	75.4 ± (7.05)
Severe LVOT calcification	9 (9.3%)

*LVOT* left ventricular outflow tract

### Procedure findings

All procedures were done by Egyptian teams with only 12% of cases proctored by non-Egyptian proctors. Table 4 summarizes the important procedure findings.

**Table 4:** Procedural details

Procedure findings	Mean ± (SD), n (%)
Anesthesia (general)	57 (59.37%)
Transesophageal echocardiography	63 (65.62%)
Percutaneous femoral route	86 (90%)
Pre-dilation	48 (50%)
Post-dilation	29 (30.2%)
Final aortic regurgitation degree	
No	53 (55.2%)
Mild	48 (50%)
Moderate	4 (4.16%)
Severe	1 (1.04%)

Transesophageal echocardiography (TEE) was frequently used (65.62%) which coincides with the increased use of general anesthesia as the primary mode of anesthesia 57 (59.37%). The trend of these ratios differs when comparing the initial experience of the first 40 cases and the last 50 cases.

The vast majority of cases were performed via the transfemoral route (90%). Alternative access sites are highlighted in Fig. 2. Balloon dilation was used in 50% of cases prior to valve implantation and 30.20% post-dilation.

**Fig. 3:**
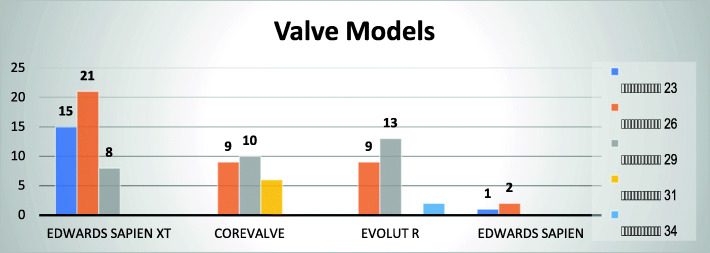
Different valves used in the study. The colors in the legend represent the sizes of the valves provided by manufacturer

The different valves and sizes used in this cohort are also summarized in Fig. 3. It is noted that the most commonly used valve type and size is the 26-mm Edwards Sapien XT valve. The Edwards Sapien XT family had the biggest share of the total valves used with 44 valves been implanted. The Corevalve which is the older version of the Medtronic® family was implanted more than the older model of the Edwards family, Sapien THV.

The percutaneous vascular closure devices were the most common closure method 79 (82.29%), followed by planned surgical closure and bail out surgical closure, in 13.5% and 4.1% of cases, respectively.

In Table 5, the percentages of complications are elaborated with vascular site injury (dissection and/or total occlusion requiring stenting or surgical repair) being the most common complication encountered and accounted for 11.5% of all cases. Thirty-day mortality occurred in 4 patients in this series, of whom three had extremely high (> 20) STS scores.

**Table 5:** Complication and mortality

Complications	n (%)
Vascular site complication	11 (11.5%)
Bailout valve in valve	2 (2.08%)
Tamponade during procedure or in-hospital	3 (3.12%)
Conversion to full sternotomy	1 (1.04%)
CVS	2 (2.08%)
Permanent pacing	7 (7.29%)
Life-threatening, disabling or major bleeding	14 (14.58%)
Mean hospital stay (days)	4.35 ± (4.9)
Median (days)	4
**IQR**	3
Hospital and 30 days mortality	4 (4.16%)

*IQR* interquartile range, *CVS* cerebrovascular

Only two patients received valve-in-valve due to migration of the valve to the aorta. Cardiac tamponade resulted mostly from the insertion of the temporary pacemaker (PM) lead. One of those patients was very obese and percutaneous pericardiocentesis was unsuccessful necessitating sternotomy and surgical evacuation of the effusion. A permanent pacemaker was implanted in 7 patients, 5 of whom received a self-expandable valve. The mean hospital stay was 4.35 days with SD of 4.9 days. The median hospital stay was 4 days.

**Fig. 4:**
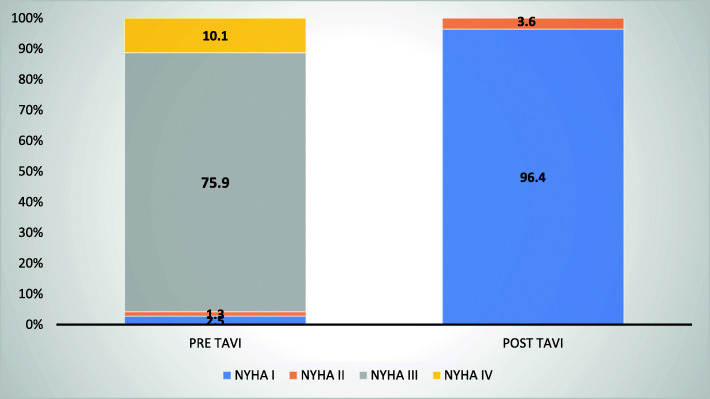
NYHA comparison pre TAVI and post TAVI

The 6-month follow-up data were available for 24 patients, while 1-year follow-up data were available for 11 patients only by a single center. Pre- and 6-month follow-up post TAVI showed a significant improvement in the functional classification of all patients (Fig. 4).

The mean Minnesota Living with Heart Failure Questionnaire (MLHF) improved significantly after the procedure (58.7 vs. 21.0, p < 0.001).

**Fig. 5:**
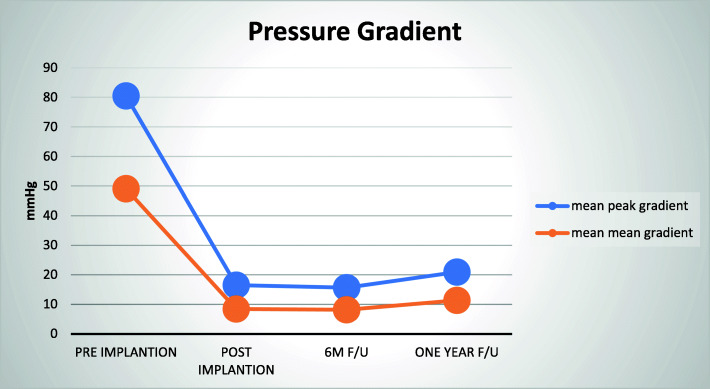
Pressure gradient follow-up

It was also noticed (Fig. 5) that the peak/mean pressure gradient has increased in the 1-year follow-up after a significant decline post-implantation. However, when comparing the 6-month follow-up and the 1-year follow-up results, it was found that there were no clinical or statistical differences between those groups.

## Discussion

To our knowledge, this is the first attempt to create an Egyptian TAVI registry. It captures the real-world experience in Egypt dealing with a new therapeutic modality that is both expensive and technically demanding. The high cost of the procedure and the lack of governmental reimbursement have resulted in a slow and limited number of patients enrolled in this registry. The preliminary data showed acceptable in-hospital and 30-day mortality of 4.16% for both. The mean age was 77.17 years and almost half of patients were females. The rate of general anesthesia and TEE were high (59.37%) due to the use of old generation valves among the retrospective patients collected.

The transfemoral approach was adopted in 89.58% of our 96 patients, compared to 74.1% in the US transcatheter valve therapy (TVT) and 74.4% in the UK registry. This could be attributed to the fact that most of the valves used in our registry were of the newer generation (Sapien XT and the Corevalve Evolut R) which have a lower profile. Moreover, the fact that TF-TAVI was found to be associated with reduced mortality encouraged all operators to attain the femoral approach as the main route of implantation [11].

An Edwards balloon-expandable valve was implanted in 48.95% of our patients and a Medtronic self-expandable device in 51.05%. In TVT registry, 76.9% of patients had balloon expandable while only 21.1% had the Medtronic Corevalve [12]. This could be attributed to the delayed introduction of the self-expandable platform in the American market. In the UK, the ratio of balloon expandable to the self-expandable is not far off our registry with 54.5% balloon expandable and 41.7% self-expandable [13].

In-hospital mortality in our study was 4.16%, which is comparable to that of the TVT and UK registries being 4.0% and 2.9%, respectively. This entails the acceptable procedural safety and health care system dealing with a new therapeutic modality that needs both trained staff and multidisciplinary management of frail and high-risk patients. In our study, not only the mortality was comparable but also the rate of complications. One of the highest encountered complications were vascular access problems 11.5% vs. 7.1% of the patient in the TVT registry and only 2.3% of the patient in the UK. This might partly be explained by the increased ratio of femoral route of performed procedures in our study. In our registry, 14.58% of the patient had life-threating, disabling, or major bleeding versus 8.4% of the patients in the TVT registry.

The need for permanent pacemaker was only 7.29% in our registry, compared to 11.8% in the TVT and 12.4% in the UK registry. This could be explained as almost half of our patient received Edwards Sapien XT valve which is known to cause less conduction disturbances, compared to other platforms, including the Lotus valve (Boston scientific transcatheter valve in 2014) which has caused conduction abnormalities in up to 30.7% of the patients requiring permanent pacemaker implantation reported in the last audit report published in 2018 by the UK registry.

In our study, we delineated the patient demographics which had significant differences than that of the western community. Major differences involved being younger in age, higher BMI, higher percentage of diabetics, and patients suffering from end stage renal disease and yet they yield a lower STS score than that of TVT and UK registries. This could be explained as our patient showed lower rates of AF and previous cardiac surgeries. This fact emphasizes the need to specific risk scores that are designed to address TAVI patients putting in consideration the frailty of such age group. As we have noticed, most of our patients were frail (*Katz index* ≤ 5 (0.2)).

Our initial experience has shown an increased use of general anesthesia (GA) approaching 70% of cases from start till end of 2016; *however*, *by the end of 2017*, *the GA dropped to 48% of cases and this was calculated in the last 36 patients*. This was achieved after building experience and abiding to minimalistic approaches. This trend is also seen in each international registry. In the UK, the use of GA declined over time dropping from 93% in 2010–2011 reaching only 20% according to their annual report published in 2018 [12]. This shift in the mode of anesthesia is supported by association in reductions in procedural inotrope requirement, intensive care unit and hospital length of stay (6.0 versus 6.5 days, P < 0.001), and combined 30-day death/stroke rates (4.8% versus 6.4%, P < 0.001) [14].

Other procedural complications (stroke, aortic regurgitation post-implantation, cardiac tamponade) were comparable to the UK and TVT registries raising no red herring. Also, the mean hospital stay across registries showed small variations, 4.35 days in our study versus 6.2 and 5.5 in the TVT and UK registry, respectively. This might be attributed that we started our program in a later stage that provided us with the newer platforms of devices that had lower profile than those used in other registries. Also, the limited number of our cohort may have also contributed to this difference.

Follow-up data was limited, as only 24 patients had completed the 6-month follow-up and 11 completed the 1-year follow-up. Those patients had improved NYHA classification, exercise tolerance, and their MLHF score was significantly better.

### Limitations and challenges

Our study included limited number of patients for statistical correlations to be deducted. Moreover, the follow-up data was only provided from a single center. The study only included patients that underwent TAVI in the 5 centers who accepted the participation in our project, while patients that underwent TAVI in different facilities were not included in this study. Another limitation was the fact that no core lab that was involved in our study and all complications were reported by the operating centers.

The high cost and availability of the valves were pivotal barriers in patient recruitment. Finally, the lack of standardized database and records in the included centers resulted in incomplete data collection and also defective follow-up data.

## Conclusion

This is the initial communication from the first TAVI registry in Egypt reporting the early outcome of 96 patients from five different centers.

TAVI outcome in Egypt appeared to be very promising with in-hospital complication, and mortality rates being comparable to international registries (4.16% vs. 4.0% in TVT registry) denoting the procedure as safe and beneficial. Establishing a national registry is critical to highlighting strength and weaknesses as well as identifying key areas for improvements.

## Supplementary Information

**Additional file 1:** Electronic Case Report Form (eCRF).

## Data Availability

The data is available upon request of the editorial board.

## Abbreviation

AS: Aortic stenosis
BMI: Body mass index
BSA: Body surface area
CT: Computed tomography
CRF: Case report form
LBBB: Left bundle branch block
LVOT: Left ventricular outflow tract
MLHF: Minnesota Living with Heart Failure Questionnaire
PVL: Paravalvular leak
QoL: Quality of life
SAVR: Surgical aortic valve replacement
SPSS: Statistical Package for Social Science
STS: Society of Thoracic Surgeons
TAVI: Transcatheter aortic valve implantation
TEE: Transesophageal echocardiography
THV: Transcatheter heart valve
VARC: Valve Academic Research Consortium
