# Supplementary material for: Early results from an Egyptian transcatheter aortic valve registry (Egy-TVR)
Source: Egypt Heart J. 2021 Jul 20;73:67. doi: 10.1186/s43044-021-00189-y (PMC8292463; doi:10.1186/s43044-021-00189-y)
Supplement: Supplementary file 1 — Additional file 1: Electronic Case Report Form (eCRF). [file 43044_2021_189_MOESM1_ESM.pdf]

MAGDI YACOUB
HEART FOUNDATION
Aswan Heart Centre

Transcatheter Heart Valve Program

Pre-procedure data

Personal Data

Name		MRN	
DOB	/ /	Age	
Gender	☐ Male ☐ Female	Hometown	
Weight	kg	Height	m
BMI	kg/m^2	BSA	m^2
Marital status	☐ Single ☐ Married ☐ Divorced ☐ Widow		
Occupation			
Phone 1		Phone 2	

Medical history and comorbidities

Critical preoperative state	☐ No ☐ Yes
Angina	☐ No ☐ Yes<br>CCS class ☐ I ☐ II ☐ III ☐ IV
SOB	☐ No ☐ Yes<br>NYHA class ☐ I ☐ II ☐ III ☐ IV
Katz Index	__ / 6
CSHA Score	☐ 1 = Very fit: robust, active, energetic, well motivated & fit; these people commonly exercise regularly & are in the most fit group for their age.<br>☐ 2 = Well: without active disease, but less fit than people in category 1<br>☐ 3 = Well, with treated comorbid disease: disease symptoms are well controlled compared with those in category 4<br>☐ 4 = Apparently vulnerable: although not frankly dependent, these people commonly complain of being "slowed up" or have disease symptoms<br>☐ 5 = Mildly frail: with limited dependence on others for instrumental activities of daily living<br>☐ 6 = Moderately frail: help is needed both with instrumental and non-instrumental activities of daily living<br>☐ 7 =Severely frail: completely dependent on others for the activities of daily living, or terminally ill
DM	☐ No<br>☐ Yes (dietary control) ☐ Yes (oral Rx)<br>☐ Yes (insulin) ☐ Yes (newly diagnosed)
HTN	☐ No ☐ Yes
Smoking	☐ Never smoked ☐ Ex-smoker ☐ Current smoker
Creatinine:	_______ mg/dL CrCl: _______ ml/min/1.73m^2
On Dialysis	☐ No ☐ Yes
Previous MI	☐ No ☐ MI <6 hours ☐ MI 6-24 hours<br>☐ MI 1-30 days ☐ MI 31-90 days ☐ MI >90 days

Pulmonary disease	☐ No ☐ COPD/emphysema<br>☐ Asthma ☐ Other: ____________________<br>FEV1: _______ % of predicted: _______<br>FVC: _______ % of predicted: _______
Liver disease	☐ No ☐ Mild ☐ Severe<br>Child Pugh Class: _______ (class C is severe)<br>MELD Score: _______ (score ≥10 is severe)<br>☐ Portocaval, spleorenal or TIPS<br>☐ Biopsy proven cirrhosis with portal hypertension or hepatocellular dysfunction<br>INR: _______ Albumin: _______ Bilirubin: _______
Neurological disease	☐ No ☐ TIA<br>☐ CVA with full recover ☐ CVA with residual defect<br>If +ve history of CVA<br>☐ Ischemic ☐ Hemorrhagic ☐ Unknown
Extracardiac arteriopathy	☐ No ☐ Yes<br>If yes<br>☐ Aortic aneurysm (history or evidence)<br>☐ >50% carotid stenosis<br>☐ Peripheral vascular disease (history, evidence or prior intervention)
Poor mobility	☐ No ☐ Yes<br>If yes<br>☐ Due to musculoskeletal disease ☐ Due to neurological dysfunction
Previous Cardiac Surgery	☐ No ☐ Yes<br>If yes<br>☐ CABG ☐ Valve surgery [specify: ________________]<br>☐ Other [specify: ________________]
Previous Intervention	Previous BAV ☐ No ☐ Yes [date:]<br>Previous TAVI ☐ No ☐ Yes [date:]<br>Previous PCI ☐ No ☐ Yes (standalone)<br>☐ Yes (part of a staged or hybrid procedure)
Psychosocial support	☐ Good ☐ Fair ☐ Poor

Magdi Yacoub
Heart Foundation
Aswan Heart Centre

ECG

☐ Sinus ☐ Afib/flutter ☐ 1st AVB ☐ RBBB ☐ LBBB

☐ CHB ☐ Paced rhythm ☐ VF or sustained VT

☐ Other abnormal rhythm [specify: ________________]

☐ Other abnormal conduction [specify: ________________]

**Notes**

MAGDI YACOUB
HEART FOUNDATION
Aswan Heart Centre

Heart Valve Team Meeting

Date:
Attended by:

STS PROM:	EuroSCORE II:

	Low Risk	Intermediate Risk	High Risk	Prohibitive Risk
**STS Score**	<4 %	4-8 %	>8 %	Risk of death or major morbidity >50% at 1-year
**Katz Index**	6/6	5/6	0-4/6	
**Major Organ System Compromise**	None	1 organ system	2 organ systems	≥3 organ systems
**Procedure Specific Impediment**	None	Possible	Possible	Severe

Katz Index of Independence in Activities of Daily Living
- Feeding, bathing, dressing, toileting, mobility, and continence

Major Organ System Compromise
- Cardiac: Severe LV systolic dysfunction, severe RV dysfunction, severe pulmonary HTN
- Pulmonary: FEV1 <50% of predicted, FVC <1.5 L
- Kidney: Estimated GFR < 60 ml/min/1.73m^2
- Liver: Any history of cirrhosis, variceal bleeding, any history of encephalopathy, INR >1.5 (in the absence of VKA use), bilirubin >2 mg/dL
- CNS: Dementia, Alzheimer's disease, Parkinson's disease, CVA with residual physical limitation
- GIT: Inflammatory bowel disease, serum albumin < 3.0 mg/dL
- Cancer: Active malignancy

Procedure-specific impediment
- Heavily calcified ascending aorta
- Chest deformity
- Patent arterial graft adherent to posterior chest wall
- Radiation damage

Overall estimated risk of SAVR ☐ **Low** ☐ **Intermediate** ☐ **High** ☐ **Prohibitive**

**MDT Decision**

MAGDI YACOUB
HEART FOUNDATION
Aswan Heart Centre

Echocardiography

LVEDD		RVD_1	
LVESD		RVD_2	
FS		RVD_3	
EF		TAPSE	
SWT “diastole”		Estimated PASP	
PWT “diastole”		TR severity	
LA AP diameter		Stroke volume	
LA volume		Indexed stroke volume	
LA volume index		Cardiac index	
RWMA			
Mitral Regurgitation	☐ No<br>☐ Mild<br>☐ Moderate<br>☐ Severe	**Aortic Regurgitation**	☐ No<br>☐ Mild<br>☐ Moderate<br>☐ Severe
**Aortic valve/root dimensions**<br><br>- Aortic Annulus<br>- Sinuses of Valsalva<br>- STJ<br>- Ascending aorta	 <br>________ mm<br>________ mm<br>________ mm<br>________ mm	**Aortic valve disease etiology**	☐ Congenital<br>☐ Degenerative<br>☐ Rheumatic<br>☐ Bioprosthetic<br>☐ Previous IE<br>☐ Other
**AV peak gradient**		**Cuspidity**	☐ Bicuspid<br>☐ Tricuspid
**AV mean gradient**			
**AV area**		**Indexed AV area**	
**Final Diagnosis and Notes**			

MAGDI YACOUB
HEART FOUNDATION
ASWAN HEART CENTRE

TAVI - CT ASSESSMENT FORM

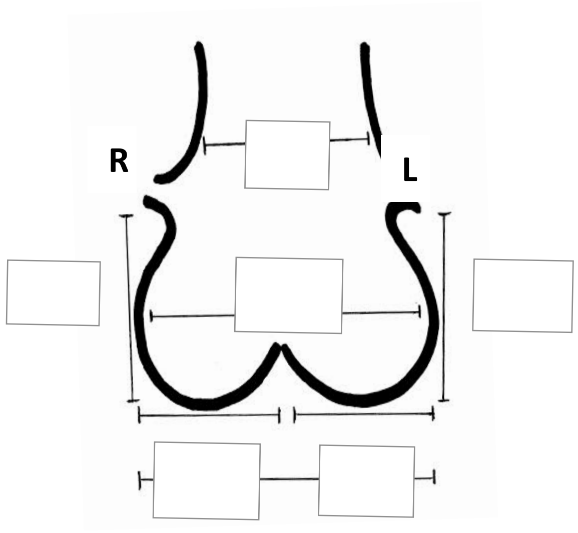

Optimal Projection	
Valve size	
Calcium score	

Coronary Artery Disease

LM	☐	%
Prox LAD	☐	%
Mid LAD	☐	%
Prox Cx	☐	%
Mid Cx	☐	%
Prox RCA	☐	%
Mid RCA	☐	%

Area	cm^3
Perimeter	mm
Min Diameter	mm
Max Diameter	mm
AD Diameter	mm
PD Diameter	mm

Apical Thrombus	☐ Yes ☐ No
LVOT calcification	☐ No ☐ Mild<br>☐ Mod ☐ Severe
Porcelain aorta	☐ Yes ☐ No
Shallow sinuses	☐ Yes ☐ No

Notes

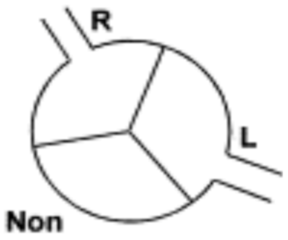

MAGDI YACOUB
HEART FOUNDATION
Aswan Heart Centre

Cardiac Catheterization

**Pressures**		
AO		
LV		
Peak-to-peak gradient		
**Coronary angiography +/- PCI**		
Vessel	% Stenosis	PCI
LMS		☐ No ☐ Yes
Proximal LAD		☐ No ☐ Yes
Mid LAD		☐ No ☐ Yes
Distal LAD		☐ No ☐ Yes
Prox LCx		☐ No ☐ Yes
Mid LCx		☐ No ☐ Yes
Distal LCx		☐ No ☐ Yes
Proximal RCA		☐ No ☐ Yes
Mid RCA		☐ No ☐ Yes
Distal RCA		☐ No ☐ Yes

Notes

MAGDI YACOUB
HEART FOUNDATION
Aswan Heart Centre

Pre-procedure laboratory investigations

CBC		Liver function tests	
Plt		AST	
WCC		ALT	
Plt		Albumin	
Renal function		Proteins	
Urea		T.Bilirubin	
Creatinine		D.Bilirubin	
Uric acid		Alkaline Phosph	
eGFR		Coagulation profile	
Electrolytes		INR	
Na		PC	
K		PTT	
Mg		Iron studies	
Ca		Ferritin	
Thyroid profile		Iron	
TSH		TIBC	
FT3		Lipid profile	
FT4		Total cholesterol	
Blood glucose		LDL	
FBS		HDL	
HbA1C		Triglycerides	

Procedure data

Date of procedure	/ /
Surgeon(s)	
Cardiologists(s)	
Proctored case	☐ No ☐ Yes
Procedure urgency	☐ Elective ☐ Urgent ☐ Emergency ☐ Salvage
Intraprocedural imaging	☐ None ☐ TTE ☐ TEE ☐ Other [specify: _______________]
Anesthesia	☐ General ☐ Other ☐ Unplanned conversion to GA
Cerebral circulation protection device	☐ No ☐ Yes
BAV prior to valve deployment	☐ Not done ☐ Completed ☐ Failed
Delivery approach	☐ Femoral - percutaneous ☐ Femoral - surgical ☐ Axillary<br>☐ Subclavian ☐ Transapical ☐ Direct aortic ☐ Carotid<br>☐ Other [specify: _______________]
Use of CPB	☐ No ☐ Yes – elective ☐ Yes – emergency
Valve manufacturer	☐ Edwards ☐ Medtronic ☐ Boston Scientific ☐ St. Jude<br>☐ Other [specify: _______________]
Valve model	☐ Sapien XT ☐ Sapien 3 ☐ Corevalve ☐ Lotus ☐ Portico<br>☐ Other [specify: _______________]
Valve serial no.	______________________________
Valve size	_____ mm
Valve failure	☐ No failure ☐ Probably iatrogenic ☐ Probably intrinsic
Vascular closure technique	☐ Manual pressure ☐ Device closure ☐ Planned surgical closure<br>☐ Bailout surgical closure (after failed percutaneous closure)

MAGDI YACOUB
HEART FOUNDATION
Aswan Heart Centre

Procedure Outcome and Complications

Valve successfully deployed	☐ No ☐ Yes<br>If no, reason:<br>☐ Access site complication ☐ Failure to negotiate iliac vessels or aorta ☐ Unable to cross aortic arch ☐ Unable to cross aortic valve<br>☐ Aborted due to vessel perforation/dissection<br>☐ Aborted due to anticipated coronary obstruction<br>☐ Aborted for other reason [specify: ________________]<br>☐ Not deployed for technical reason<br>☐ Other failure to deploy [specify: ________________]
Post deployment AV peak gradient	_____ mmHg
Post deployment AV mean gradient	_____ mmHg
AR at end of procedure	☐ None ☐ Mild ☐ Moderate ☐ Severe ☐ Unknown
AR evaluated by	☐ Angiograph ☐ TEE ☐ TTE
Valve malpositioning	☐ None ☐ Valve migration ☐ Valve embolization<br>☐ Ectopic valve deployment
Bailout valve-in-valve	☐ No ☐ emergency during index procedure<br>☐ Non-emergency during index procedure for suboptimal result
Post-implantation balloon dilatation	☐ No ☐ Yes ☐ Not applicable
Further valve Intervention (not during index procedure but before discharge	☐ None ☐ TAVI ☐ Surgical AVR ☐ BAV<br>☐ Intervention on another valve ☐ Other [specify: ________________]
Tamponade during/post procedure	☐ No ☐ Yes (requiring surgical intervention)<br>☐ Yes (requiring percutaneous intervention
Major apical cannulation complications	☐ No ☐ Yes

Conversion to full sternotomy	☐ No ☐ Yes
Bailout PCI	☐ No ☐ Yes
Periprocedural MI	☐ No ☐ Yes
Permanent pacing	☐ No ☐ Yes-preprocedure therapeutic (including distant past) ☐ Yes - pre-procedure prophylactic ☐ Yes - per-procedure ☐ Yes - post-procedure
CVA	☐ No ☐ Yes - Ischemic ☐ Yes - hemorrhagic ☐ Yes - undetermined
If yes, modified Rankin Score at 90 days	______
Vascular access site related complications	☐ No ☐ Major ☐ Minor
Percutaneous closure device failure	☐ No ☐ Yes ☐ Not applicable
Bleeding	☐ No ☐ Yes – life threatening ☐ Yes – major ☐ Yes – minor
No of units of blood transfused	______
AKI within 7 days of procedure	☐ No AKI ☐ Stage 1 ☐ Stage 2 ☐ Stage 3 ☐ Unknown
New RRT up to discharge	☐ No ☐ Yes
Death to hospital discharge	☐ No ☐ Yes
Date of discharge or death	/ /
Drugs at discharge - antithrombotic	☐ None ☐ Warfarin ☐ Dabigatran ☐ Rivaroxaban ☐ Apixaban ☐ Other [specify: ________________]
Drugs at discharge - antiplatelet	☐ None ☐ Aspirin ☐ Clopidogrel ☐ Prasugrel ☐ Ticagrelor ☐ Other [specify: ________________]

MAGDI YACOUB
HEART FOUNDATION
Aswan Heart Centre

Follow up

Follow up at 1 year

Angina	☐ No	☐ Yes			
	CCS class	☐ I	☐ II	☐ III	☐ IV
SOB	☐ No	☐ Yes			
	NYHA class	☐ I	☐ II	☐ III	☐ IV

Follow up at 3 years

Angina	☐ No	☐ Yes			
	CCS class	☐ I	☐ II	☐ III	☐ IV
SOB	☐ No	☐ Yes			
	NYHA class	☐ I	☐ II	☐ III	☐ IV

Late valve stenosis	☐ No ☐ Yes
Date of Dx of significant stenosis	/ /
Late intrinsic valve regurgitation (not paravalvular)	☐ No ☐ Yes
Date of Dx of clinically significant regurgitation	/ /
Valve failure mode	☐ Stent creep ☐ Pannus ☐ Calcification<br>☐ Support structure deformation ☐ Prosthesis-patient mismatch<br>☐ Endocarditis ☐ Valve thrombosis<br>☐ Native leaflet impending prosthetic motion ☐ malposition (too high/too low) ☐ Leaflet wear/tear/perforation ☐ Leaflet malcoaptation
Late paravalvular regurgitation	☐ No ☐ Yes – new<br>☐ Yes – unchanged from existing ☐ Yes – increased from existing<br>☐ Yes – less than post-porcedure
Date of Dx of clinically significant paravalvular regurgitation	/ /
